# A Quantitative Study on the Ethnobotanical Knowledge about Wild Edible Plants among the Population of *Messiwa*

**DOI:** 10.4314/ejhs.v32i6.22

**Published:** 2022-11

**Authors:** Ridwane Ghanimi, Ahmed Ouhammou, Rachid Ait Babahmad, Mohamed Cherkaoui

**Affiliations:** 1 Laboratory of Pharmacology, Neurobiology, Anthropobiology, Environment and, Behaviour, Department of Biology, Faculty of Sciences Semlalia, Cadi Ayyad University, Marrakech, BP 2390, 40000, Morocco; 2 Laboratory of Microbial Biotechnologies, Agrosciences and, Environment (BioMAgE), Agrosciences, Phytob iodiversity and Environment team, regional herbarium ‘MARK', Department of Biology, Faculty of Sciences Semlalia, Cadi Ayyad University, Po. Box 2390, Marrakech, 400001, Morocco

**Keywords:** Ethnobotany, wild edible plants, Traditional knowledge, Erosion, Quantitative study

## Abstract

**Background:**

The preservation of traditional knowledge of wild edible plants (WEPs) is one of the challenges to the sustainability of natural resources. Therefore, it is crucial to assess the traditional knowledge of WEPs in relation to some socio-demographic and economic factors.

**Methods:**

The survey was conducted among the Messiwapopulationthrough a semi-structured questionnaire. The first part of the questionnaire concerns socio-demographic and economic information, while the second part concerns the plants recognized and used by the informant. The recognition frequency (RF), the use frequency (UF), the general consumption frequency(CF), the recent consumption frequency (RCF), and the correlation between these frequencies were evaluated. A comparison of means was also used to compare informant's knowledge according to their socio-demographic and economic status.

**Results:**

The three species;Foeniculum vulgare, Ziziphus lotus, and Malva sylvestris were the most recognized (FR = 1) and the most used (FU = 1). The consumption frequency (CF) and the recent consumption frequency (RCF) for Foeniculum vulgare were 1 and 0.9, respectively. Taraxacum getulum, Calendula arvensis and Cyperus rotundus were the least recognized (FR= 0.16; 0.16; 0.48) and least used (FU = 0.3; 0.3; 0.4) species, respectively. The informants who showed a high level of traditional knowledge on WEPs were housewives, with a low level of schooling and at least 45 years old.

**Conclusion:**

Despite the decline in traditional knowledge about wild edible plants, some populations preserve this knowledge, especially among the elderly. Therefore, documentation of this knowledge is necessary through ethnobotanical and ethnomedicinal studies.

## Introduction

Wild edible plants (WEPs) are natural resources that include nutrition, therapy, aesthetics, and spiritual purposes (1,2,3). The use of these wild edible plants has been very important in Morocco as in many places around the world (2,4). In particular during the periods of dearth where the people turned to these plants, despite the toxicity of some of these species (2,5).

The consumption of WEPs has shown a worrying decline in the last few years (6,7). Modernization, climate change, and excessive consumption of a limited number of domesticated species have been among the causes of this regression (8). Ignorance of these wild species by the descendant and their progressive lack in the local markets are two other factors that may further accelerate this regression (9,10).

The region of Al-Haouz in Morocco is known for its plant biodiversity and the presence of some Moroccan endemic plants like *Lavandula mairei* Humbert (11,12). Moreover, the population of the Al-Haouz region mainly the Messiwa, which is an original population, still uses some wild edible plants in traditional dishes (11).

The present study aimed to assess the knowledge of the *Messiwa* population regarding some wild edible plants that were used in the Al-Haouz region. The difference in knowledge among the respondents was also assessed according to their socio-demographic and economic status.

## Methods

**Figure 1:**
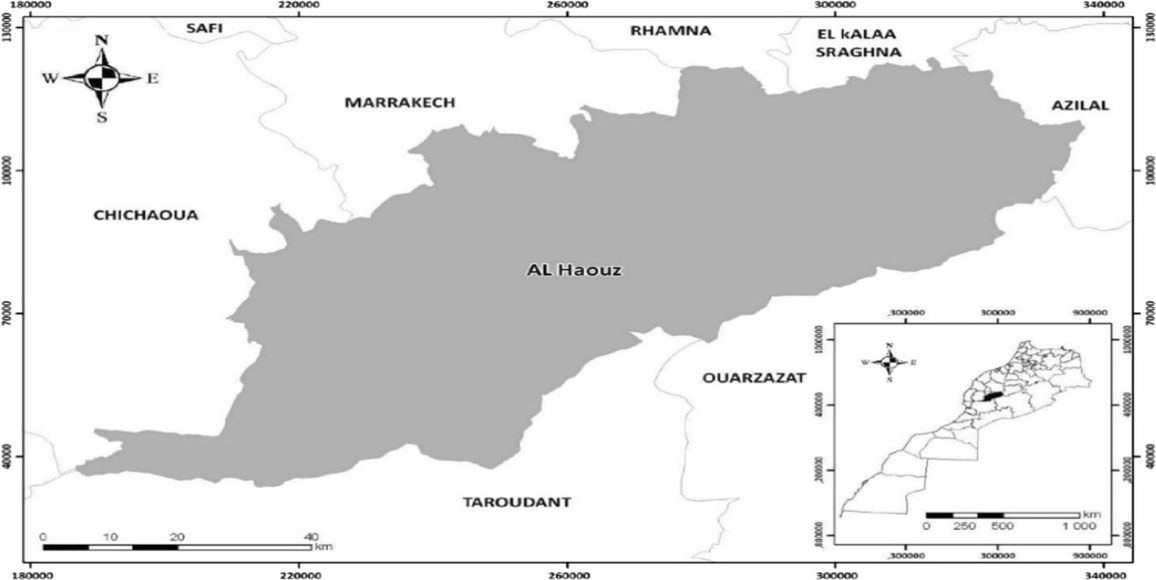
The geographical location of the study area.

**Study area**: This study was conducted in the province of Al-Haouz in Morocco (Figure 1) among the *Messiwa* population. The topography of the Al-Haouz region shows a plain part and a mountainous part (belonging to the High Atlas Mountains) (13). The rural communities represent 88% of the total population of Al-Haouz, with farming and pastoral activities for a large part of this population(14). Moreover, as this region includes a plain part and another mountainous part, the climate ranges from arid to humid. Furthermore, this diversity of climate is accompanied by a diversity of vegetation (15).

**The studied population**: The *Messiwa* population is a native population of the Al-Haouz region (16). Regarding the activities of the local population, a part of them is involved in agricultural activities such as subsistence farming, nurseries, and herbal plants. The other part has non-agricultural occupations such as private security, local trade, and construction work. However, the majority of women are housewives (14). All informants were adults able to answer our questions. Informed consent was obtained from all informants after explaining the purpose of the study.

**Questionnaire design**: The questionnaire included two parts; the first part contained the respondent's personal information (age, gender, family types, income, educational level, occupation...) and the second part represented a list of 64 wild edible plants cited by the *Messiwa*population in a previous study by Ghanimi et al. 2022 (11). The herbarium containing the 64 WEPs was represented to the informants to determine which species they would recognize, then specify whether the recognized plant is edible, determine if they have ever consumed this plant, and the last time of use (within 2 years or more).

**Recognition frequency (RF)**: The recognition frequency of the plant represents the number of informants recognizing the plant from the herbarium. The RF was calculated by the following formula:

$$\mathrm{RF}=\frac{\text{Number of informants who recognized the plant}}{\text{total number of informants}}$$

**Use frequency (UF)**: The use frequency of the plant represents the frequency of people considering the plant edible and it was calculated by the following formula:

$$\mathrm{UF}=\frac{\text{Number of informants considering the plant as edible}}{\text{Total number of informants}}$$

**General consumption frequency (CF)**: General consumption frequency represents the frequency of people who have previously consumed the plant. The CF was calculated as:

$$\mathrm{CF}=\frac{\text{Number of informants who consumed the plant}}{\text{total number of informants}}$$

**Recent Consumption Frequency (RCF)**: The recent consumption frequency represents the frequency of people who have consumed the plant during the last two years. The RCF was calculated by the following formula:

$$\mathrm{RCF}=\frac{\text{Number of informants who have used the plant in the last 2 years}}{\text{total number of informants}}$$

**Statistical analysis**: The collected data were analyzed using IBM SPSS 20 software and Excel 2010. Analysis of variance (ANOVA) was used followed by the Duncan test. The Student test was also used and the difference was considered significant at P < 0.05.

## Results

**Structure of the studied population**: The number of respondents was 200 (Table 1). Among them were 50 women aged 45 or more, 50 women under 45, 50 men aged 45 or more, and 50 men under 45. More than half of the men work in agriculture (52%), while the majority of the women are housewives (89%). Families consisting of parents and children (single family) represent 79% and extended families (containing children, parents, and grandparents) represent only 21%. Regarding monthly income, 65% of men and 75% of women have incomes below 3000 MDh (equivalent to 300 €). In addition, women have a high illiteracy rate (75%) compared to men who showed 48%. The percentage of people who have a university level was very low for women (1%) and also for men (7%).

**Table 1:** Socio-demographic and economic structure of the study population

Variables		Man (%)	Women (%)
**Age**	Less than 45 years old	50	50
	45 years and older	50	50
**Occupation**	Agricultural	52	6
	Non-agricultural	48	5
	Housewife	0	89
**Family type**	Simple	79	79
	Extended	21	21
**Income**	Less than 3000 MDh	65	75
	3000 to 5000 MDh	25	20
	More than 5000	10	5
**Education level**	Illiterate	48	75
	Primary	20	14
	Middle school	18	9
	High School	7	1
	University	7	1

**Quantitative indices**: Table 2 represents the list of edible wild plants and the different quantitative indices (RF, UF, CF, and RCF). The three species; *Foeniculum vulgare*, *Ziziphus lotus*, and *Malva sylvestris*were the most recognized plants by the informants. The recognition frequency (RF) and use frequency (UF) for the three plants were 1; which means that all the informants were able to recognize these three species and they knew that they are edible. Regarding the general consumption frequency; *Foeniculum vulgare* showed an FCR = 1; thus indicating that all informants have consumed this species before. While, *Ziziphus lotus* and *Malva sylvestris* were consumed at least once by 99% and 97% of informants, respectively. On the other hand, the highest recent consumption frequency (RCF)was observed in *Foeniculum vulgare*(RCF= 0.9), which indicates that 90% of informants consumed the plant in a period not exceeding the last two years.

**Table 2:** List of wild edible plants, their medicinal uses, and their recognition, use, and consumption indices

Scientific name	Family	Local name	Medicinal uses	FR	FU	FC	FCR
*Ajuga iva* (L.) Schreb	Lamiaceae	Chandgoura	cold and abdominal pain	0,36	0,1	0,08	0,06
*Allium roseum* L	Amaryllidaceae	lbsal barri	only edible in times of famine	0,48	0,31	0,23	0,13
*Arbutus unedo* L	Ericaceae	Sasnou	only edible	0,43	0,41	0,38	0,22
*Arisarum vulgare* O.Targ.Tozz	Araceae	Irni	only edible	0,26	0,14	0,07	0,04
*Aristolochia paucinervis* Pomel	Aristolochiaceae	Brztm	only edible	0,38	0,21	0,12	0,05
*Artemisia herba-alba* Asso	Compositae	Chih	Aromatizer, abdominal pain, and wounds	0,89	0,54	0,47	0,35
*Asparagus albus* L	Asparagaceae	Hmissou	Cold	0,63	0,61	0,48	0,27
*Asparagus altissimus* Munby	Asparagaceae	Hmissou	Cold	0,63	0,61	0,48	0,27
*Asparagus horridus* L	Asparagaceae	Hmissou	Cold	0,63	0,61	0,48	0,27
*Calendula arvensis* M.Bieb	Compositae	Jamra	Only edible	0,16	0,03	0,01	0
*Capparis spinosa* L	Capparaceae	Kabbar	Rheumatism and cold	0,4	0,33	0,22	0,11
*Caralluma europaea* (Guss.) N.E.Br	Apocynaceae	Ddaghmous	Diabetes and cough	0,45	0,36	0,17	0,08
*Carlina gummifera* (L.) Less	Compositae	Addad	only edible	0,56	0,09	0,05	0
*Ceratonia siliqua* L	Leguminosae	Kharoub	Good for stomach	0,94	0,94	0,93	0,61
*Chamaerops humilis* L	Arecaceae	Doum	only edible	0,46	0,09	0,05	0
*Cistus creticus* L	Cistaceae	Irgual	cold, appetizer	0,14	0,1	0,08	0,03
*Cistus salviifolius* L	Cistaceae	Irgual	cold, appetizer	0,14	0,1	0,08	0,03
*Cladanthus arabicus* (L.) Cass	Compositae	Aourzid	Good for stomach and anemia	0,38	0,18	0,07	0,03
*Cynara cardunculus* L	Compositae	khrchouf lbaldi	only edible	0,78	0,77	0,71	0,45
*Cynodon dactylon* (L.) Pers	Poaceae	Njem	only edible	0,77	0,1	0,03	0,02
*Cyperus rotundus* L	Cyperaceae	Tamoussayt	good for the hair	0,48	0,04	0,01	0
*Diplotaxis* sp.	Brassicaceae	Bohmmou	only edible	0,31	0,19	0,12	0,06
*Drimia maritima* (L.) Stearn	Asparagaceae	Igufil	only edible	0,3	0,09	0,04	0,01
*Dysphania ambrosioides* (L.)	Amaranthaceae	Mkhinza	Fever	0,92	0,86	0,8	0,5
Mosyakin&Clemants							
*Emex spinosa* (L.) Campd	Polygonaceae	Hommida	only edible	0,58	0,35	0,26	0,1
*Foeniculum vulgare* Mill	Apiaceae	Besbas	Aromatizer and good for stomach and the digestion	1	1	1	0,9
*Glaucium corniculatum* (L.) Curtis	Papaveraceae	Hbbosousou	only edible	0,32	0,3	0,26	0,1
*Glebionis coronaria* (L.) Cass. ex Spach	Compositae	Guhouan	calming and relaxing	0,43	0,35	0,24	0,1
*Herniaria hirsuta* subsp. cinerea (DC.) Cout	Caryophyllaceae	hrrast lahjar	good for kidney stones	0,53	0,49	0,3	0,14
*Juncus acutus* L	Juncaceae	Essmar	only edible	0,5	0,15	0,07	0,02
*Lathyrus clymenum* L	Leguminosae	Ikikr	only edible	0,47	0,25	0,15	0,05
*Lavandula dentata* L	Lamiaceae	Halhal	cold, abdominal pain	0,38	0,28	0,14	0,06
*Lavandula mairei* Humbert	Lamiaceae	Guorzghial	Cold	0,34	0,27	0,17	0,05
*Lavandula stoechas* L	Lamiaceae	Khzama	Cold and good for the urinary tract	0,87	0,73	0,55	0,33
*Malva sylvestris* L	Malvaceae	Khobbiza	only edible	1	1	0,97	0,85
*Mentha pulegium* L	Lamiaceae	Fluo	Aromatizing, cold, cooling and refreshing	0,93	0,92	0,91	0,81
*Mentha rotundifolia* (L.) Huds	Lamiaceae	timijja lmanta	Aromatizing, cooling, abdominal pain and refreshing	0,67	0,63	0,56	0,39
*Mentha suaveolens* Ehrh	Lamiaceae	timijja nwaman	Aromatizing, cooling, abdominal pain and refreshing	0,94	0,9	0,85	0,7
*Mercurialis annua* L	Euphorbiaceae	hourrigua lmalsa	Cooling	0,52	0,28	0,14	0,05
*Morus alba* L	Moraceae	Tût	only edible	0,92	0,92	0,9	0,62
*Nasturtium officinale* R.Br	Brassicaceae	Gurnounch	cold, and back pain	0,4	0,36	0,3	0,07
*Olea oleaster* Hoffmanns. & Link	Oleaceae	Jbouj	only edible	0,64	0,31	0,16	0,08
*Ononis natrix* L	Leguminosae	Afzdad	Anemia	0,26	0,12	0,07	0,02
*Opuntia ficus-indica* (L.) Mill	Cactaceae	Handia	only edible	0,95	0,95	0,95	0,7
*Papaver rhoeas* L	Papaveraceae	Bellaaman	measles and fever	0,78	0,45	0,29	0,15
*Peganum harmala* L	Nitrariaceae	Harmal	cold, abdominal pain and fumigation	0,67	0,21	0,13	0,08
*Phoenix dactylifera* L	Arecaceae	Ablouh	only edible	0,95	0,95	0,93	0,72
*Portulaca oleracea* L	Portulacaceae	Trejla	only edible	0,97	0,97	0,94	0,84
*Quercus ilex* L	Fagaceae	Ballout	only edible	0,93	0,93	0,92	0,75
*Ridolfia segetum* (L.) Moris	Apiaceae	Tabch	liver disease	0,36	0,25	0,14	0,05
*Rosa canina* L	Rosaceae	Tighfrt	only edible	0,41	0,28	0,2	0,02
*Rosmarinus officinalis* L	Lamiaceae	Azir	Aromatizer, abdominal pain, cold	0,95	0,86	0,8	0,62
*Rubia peregrina* L	Rubiaceae	Lfoua	Anemia	0,76	0,73	0,61	0,4
*Rubus ulmifolius* Schott	Rosaceae	Achddir	only edible	0,36	0,28	0,19	0,11
*Rumex pulcher* L	Polygonaceae	Selk	only edible	0,51	0,45	0,4	0,25
*Scolymus hispanicus* L	Compositae	Guernina	only edible	0,81	0,79	0,67	0,4
*Silene vulgaris* (Moench) Garcke	Caryophyllaceae	Taghighacht	only edible	0,27	0,05	0,01	0,01
*Taraxacum getulum* Pomel	Compositae	Jamra	only edible	0,16	0,03	0,01	0
*Tetraclinis articulata* (Vahl) Mast	Cupressaceae	Aaraar	abdominal pain and on wounds	0,67	0,33	0,19	0,08
*Thymus saturejoides* Coss	Lamiaceae	Zaatar	Aromatizer, abdominal pain	0,98	0,95	0,92	0,82
*Thymus willdenowii* Boiss	Lamiaceae	Zaaitra	Aromatizer, abdominal pain	0,9	0,89	0,86	0,72
*Urtica dioica* L	Urticaceae	hourrigua lharcha	urinary pain, stomach and cold	0,79	0,26	0,16	0,06
*Ziziphus lotus* (L.) Lam	Rhamnaceae	Nbag	Good for the stomach and intestines	1	1	0,99	0,86

The two species, *Taraxacum getulum*, and *Calendula arvensis,* were respectively the least recognized (FR= 0.16) and the least used (FU = 0.3). The percentage of people who have consumed these two species at least once before was 1%, while none of our informants have consumed these two plants in the last two years (FR = 0).

**Correlations among the four frequencies**: The correlation analysis between the four frequencies (FR, FU, FC and FCR) was performed (Pearson correlation coefficient, r). Table 3 shows that the correlations between the different frequencies were all significant at the 0.01 level. The strongest correlation was observed between the consumption frequency and the use frequency (r = 0.990). This indicates that 99% of those who recognized the plant as edible had consumed it at least once before.

**Table 3:** The correlation between the frequencies of recognition, use, general consumption and recent consumption

	RF	UF	CF	RCF
**RF**	1	0.865**	0.850**	0.843**
**UF**		1	0.990**	0.960**
**CF**			1	0.981**
**RCF**				1

** The correlation is significant at the 0.01 level

**Comparison of means** (table 4): The difference in means between women and men regarding the number of wild edible plants recognized was significant at the 0.05 level. The mean for women was 38.96 ±10.67, while men showed a mean of 34.85 ±11.65. For the age category, the difference of means was highly significant in favor of those aged 45 years and over (41.38±10.73). In addition, those working in nonagricultural fields recognized fewer wild edible plants than housewives and people working in agricultural fields. The educational level also showed a highly significant difference between people with low educational levels (illiterate and people with primary levels) and people with relatively high academic levels (middle school, high school, or university). In contrast, family type and income did not show significant differences.

**Table 4:** Comparison of averages by socio-demographic and economic status

Variables	Number	Average recognized plants	Statistical test	Homogeneous groups
**Sex**				
Men	100	34,85 ±11,65	t= -2.6 *	
Women	100	38,96 ±10,67		
**Age**				
Less than 45 years	100	32,43±10,14	t= -6.062 ***	
45 years and over	100	41,38±10,73		
**Occupation**				
Agricultural	58	37,64±11,44		
Non-agricultural	52	32,98±11,62	F= 4.517 *	(1,3)
Housewife	90	38,70±10,66		
**Education level**				
Illiterate Primary	123	39,94±10,59		
college level	34	34,80±09,41	F= 11.967 ***	(1,2)(3, 4,5)
Middle school	27	34,67±11,25		
High school	8	21,00±05,24		
University	8	22,75±07,99		
**Family type**				
Simple	158	36,13±11,28	t= -1.88 ns	
Extended	42	39,81±11,21		
**Income**				
Less than 3000 MDh	140	37,37±11,49		
3000 to 5000 MDh	45	35,71±10,55	F= 4 ns	
More than 5000 MDh	15	36,13±12,61		

T = student test of comparison of 2 means; F = Fisher test of analysis of variance

* test significant at the 5% level

ns = not significant, in brackets means that the means are equal

## Discussion

*Foeniculum vulgare* is a species widely consumed in Mediterranean countries (11,17,18). This species is known for its benefits on digestion and the function of the gastroenteric system. The other two species; *Ziziphus lotus* and *Malva sylvestris*, are known mainly for their nutritional use and they are among the most cited species in Morocco through many ethnobotanical studies, which could explain their high recognition and consumption frequencies (2,3,11,19).

The recognition of a wild plant as an edible species is strongly related to its consumption. Therefore, the consumption of wild edible plants represents several benefits; such as diversification of the nutrient resources and the development of the local economy (20,21). The valorization of these natural resources is of crucial importance in light of strategies that aim to respect biodiversity and prevent malnutrition in developing countries (22,23). Moreover, the promotion of these wild edible species can represent a source of food supplements, new therapeutic molecules, oils, and natural cosmetic products.

The sociodemographic characteristics of communities strongly influence their knowledge and interactions with the environment (24,25). The erosion of wild plant knowledge has been reported by several authors and it has been influenced by different variables, the most important of which are age and gender (26–28). The study conducted on the population of El-Jadida by Tbatou, Belahyan, and Belahsen (2016) and the study conducted by Ghanimi et al. (2022) in the Al-Haouz region have shown that older women have significant ethnobotanical knowledge about wild edible plants more than other people(11,29). This difference in favor of older women could be due to the traditional lifestyle of these women in addition to their preference to take herbal treatments instead of using pharmacy products (11,30).

This study is another warning signal to protect biodiversity by promoting these wild edible plants and documenting this knowledge among people who show a high level of traditional knowledge, especially in our case among elderly women.

In conclusion, wild edible plants occupy an important place due to their nutritional and therapeutic potential. The most appreciated species by the population of *Messiwa* was *Foeniculum vulgare*. On the other hand, it was observed that the people who have a high level of knowledge about WEPs were housewives, aged 45 years or more and with a low level of education. This trend is in favor of a modern lifestyle, which is beginning to replace increasingly traditional life. Therefore, this work can be the basis for other similar surveys to evaluate the erosion of knowledge which is in continuous decline.
